# Improving l-threonine production in *Escherichia coli* by elimination of transporters ProP and ProVWX

**DOI:** 10.1186/s12934-021-01546-x

**Published:** 2021-03-02

**Authors:** Shuaiwen Wang, Yu Fang, Zhen Wang, Shuyan Zhang, Liangjia Wang, Yong Guo, Xiaoyuan Wang

**Affiliations:** 1 State Key Laboratory of Food Science and Technology, Jiangnan University, 1800 Lihu Avenue, Wuxi, 214122 China; 2 International Joint Laboratory on Food Safety, Jiangnan University, Wuxi, 214122 China; 3 Key Laboratory of Industrial Biotechnology, School of Biotechnology, Ministry of Education, Jiangnan University, Wuxi, 214122 China

**Keywords:** *Escherichia coli*, l-Threonine production, Betaine biosynthesis, Osmotic pressure, *proP* and *proVWX*

## Abstract

**Background:**

Betaine, an osmoprotective compatible solute, has been used to improve l-threonine production in engineered *Escherichia coli* l-threonine producer. Betaine supplementation upregulates the expression of *zwf* encoding glucose-6-phosphate dehydrogenase, leading to the increase of NADPH, which is beneficial for l-threonine production. In *E. coli*, betaine can be taken through ProP encoded by *proP* or ProVWX encoded by *proVWX.* ProP is a H^+^-osmolyte symporter, whereas ProVWX is an ABC transporter. ProP and ProVWX mediate osmotic stress protection by transporting zwitterionic osmolytes, including glycine betaine. Betaine can also be synthesized in *E. coli* by enzymes encoded by *betABIT*. However, the influence of ProP, ProVWX and *betABIT* on l-threonine production in *E. coli* has not been investigated.

**Results:**

In this study, the influence of ProP, ProVWX and *betABIT* on l-threonine production in *E. coli* has been investigated. Addition of betaine slightly improved the growth of the l-threonine producing *E. coli* strain TWF001 as well as the l-threonine production. Deletion of *betABIT* retarded the growth of TWF001 and slightly decreased the l-threonine production. However, deletion of *proP* or/and *proVWX* significantly increased the l-threonine production. When *proP* was deleted, the l-threonine production increased 33.3%; when *proVWX* was deleted, the l-threonine production increased 40.0%. When both *proP* and *proVWX* were deleted, the resulting strain TSW003 produced 23.5 g/l l-threonine after 36 h flask cultivation. The genes *betABIT*, *proC*, *fadR*, *crr* and *ptsG* were individually deleted from TSW003, and it was found that further absence of either *crr* (TWS008) or *ptsG* (TWS009) improved l-threonine production. TSW008 produced 24.9 g/l l-threonine after 36 h flask cultivation with a yield of 0.62 g/g glucose and a productivity of 0.69 g/l/h. TSW009 produced 26 g/l l-threonine after 48 h flask cultivation with a yield of 0.65 g/g glucose and a productivity of 0.54 g/l/h, which is 116% increase compared to the control TWF001.

**Conclusions:**

In this study, l-threonine-producing *E. coli* strains TSW008 and TSW009 with high l-threonine productivity were developed by regulating the intracellular osmotic pressure. This strategy could be used to improve the production of other products in microorganisms.

## Introduction

In the process of fermentation production, we often encounter the growth restriction and the termination of product biosynthesis due to the osmotic pressure imbalance. Therefore, improving the ability of bacteria to tolerate osmotic stress or using special strains that are resistant to hypertonicity become a new direction for fermentation production of biological products such as l-threonine, lactic acid, acetic acid, l-lysine, and pyruvate [1]. The hypertonic-resistant strain *Halomonas bluephagenesis* has been developed to produce l-threonine under high osmotic pressure, by deleting the l-threonine importer (*sstT*) and l-threonine dehydrogenase (*thd*), and expressing the genes thrA*BC, lysC*and *rhtC* relevant to the biosynthesis and export of l-threonine. The resulting recombinant strain produced 33 g/l l-threonine when grown under open unsterile conditions with a productivity of 1.368 g/l/h in fed-batch fermentation [2]. Osmoprotectants have been used to improve the growth and yield limitations caused by the imbalance of osmotic pressure during the fermentation process [3, 4]. Betaine has been applied as one of the osmoprotectants due to its special methylation-related structure [3, 5, 6]. In *E. coli*, betaine plays an indispensable role in cell growth and production of target metabolites [5]. To maintain the activity of the bacteria in the later stage of fermentation, betaine can be added to improve the tolerance to osmotic pressure [7]. Addition of betaine can increase the production of lactic acid or l-threonine in *E. coli* [5] as well as the production of lysine in *Corynebacterium glutamicum*. A l-threonine producing *E. coli* JLTHR was cultured in fermentation medium containing 20 g/l glucose on fed-batch fermentation and explored the effect of betaine on l-threonine production. The maximum l-threonine production of 127.3 g/l was obtained with 58.12% glucose conversion percentage [4]. Since hypertonic tolerant strains need complex screening and genetic modification, modification of the existing high-yielding bacteria to improve their osmotic resistance is a good strategy [8, 9].

The betaine biosynthesis in *E. coli* involves: choline dehydrogenase (*betA*), betaine aldehyde dehydrogenase (*betB*), choline: H(+) symporter (*betT*), and a regulatory gene *betI* [10]. These genes can be induced by choline, oxygen, and osmotic stress [11, 12]. Under low osmotic pressure, the balance of osmotic pressure inside and outside the cell is maintained by potassium ions entering the cell through the sodium and potassium pump. When the extracellular osmotic pressure rises, the osmotic protective agent such as betaine accumulates in the cell either by the absorption from the media through transport proteins or by the intracellular biosynthesis. Glycine betaine is more effective than other osmolytes for increasing osmolality in water or concentrated protein solutions in vitro [13]. The major betaine transport proteins in *E. coli* are the primary transporter ProP and the secondary transporter ProVWX which are regulated by osmotic pressure [14]. These transporters also play a role on transporting other osmoprotectants such as proline and ectoine [6, 15]. ProP consists of a single polypeptide embedded in the cytoplasmic membrane [16]. ProVWX in *E. coli* has a very high affinity for glycine betaine, it is a multi-component and binding-protein-dependent transport system. Both *proP* and the three genes *proV, proW and proX* are coordinately expressed under the control of one major osmo-regulated promoter [15]. For example, *E. coli* can survive in human urine and in the murine bladder due to *proP* expression [17], resulting in urinary tract colonization. Transcriptomics analysis of *E. coli* under different osmotic pressures or after the addition of osmoprotectants such as betaine [18] showed that the transcription levels of *proP* and *proVWX* rapidly increase under high osmotic pressure. ProP and ProVWX are also responsible for the transport of proline, and proline also bears part of the osmotic protection function. Therefore, it is very important to eliminate the interference of proline on the research of betaine. The gene *proC* encodes a pyrroline-5-carboxylate reductase for proline synthesis [19].

In *E. coli*, glucose is converted to pyruvate through the glycolysis pathway, and pyruvate is converted to acetyl-CoA to introduce carbon flow into the tricarboxylic acid (TCA) cycle [20]. Oxaloacetate, an important intermediate in the TCA cycle, is synthesized from phosphoenol pyruvate (PEP) by phosphoenolpyruvate carboxylase (*ppc*) [20]. Aspartate aminotransferase (*aspC*) converts oxaloacetate to l-aspartic acid. In the l-threonine-producing *E. coli* strain TWF001, the l-threonine synthesis pathway is strengthened, the key genes related to NAD(P)H regeneration are up-regulated, and many genes related to glycolysis and TCA cycle are down-regulated [21]. In previous studies, a variety of methods have been tried to increase the l-threonine production in *E. coli* TWF001. When expression of seven genes (*iclR*, *aspC*, *arcA*, *cpxR*, *gadE*, *pykF*, *fadR* and *aspC*) in TWF001 was regulated by replacing their original RBS sequence with the *thrL* leader regulatory elements, 26.50 g/l l-threonine was produced from 40 g/l glucose [22]. When the PHB synthesis pathway was introduced into TWF001, a significant increase of l-threonine production as well as the PHB formation were obtained. TWF001/pFW01-*phaCAB* could produce 96.4 g/l l-threonine in 3 l fermenter and 133.5 g/l l-threonine in 10 L fermenter, respectively [23]. When *arcA*, *iclR* and *tdcC* were deleted in TWF001, l-threonine production reached 26 g/l, which is a 109.7% increase compared to the control TWF001 [24]. When a thermal switch system was designed and applied to rebalance carbon substrates between pyruvate and oxaloacetate, l-threonine yield in TWF001 was significantly increased and resulting in the highest l-threonine yield of 124.03%, which exceeds the best reported yield (87.88%) and the maximum available theoretical value of l-threonine production (122.47%). [25].

In *E. coli*, FadR regulates fatty acid metabolism by inhibiting the transcription of *fadL*, *fadD*, *fadE*, *fadH*, *fadM*, *fadBA*, and *fadIJ* operons related to fatty acid degradation; however, when it binds to coenzyme A, this inhibition can be eliminated. Therefore, in the absence of FadR, the fatty acid degradation would enhance and the lipid biosynthesis would be weakened in *E. coli* [20]. *E coli* cells absorb glucose through GalP, MglABC, or the phosphotransferase system (PTS). GalP or MglABC simply transports glucose through the membrane, but PTS converts glucose to glucose-6-P during the transport process, and the phosphate group is provided by PEP through a series of phosphorylation. When PEP is needed, blocking the PTS system to preserve PEP is a good choice [26].

In this study, the influence of osmotic pressure on l-threonine production in TWF001 was investigated by changing the expression levels of *proP*, *proVWX*, *betABIT, proC*, as well as some key genes related to l-threonine biosynthesis.

## Methods

### Bacteria strains and culture conditions

The strains used in this study are listed in Table 1. *E. coli* strains were usually cultured in LB medium (10 g/l tryptone, 10 g/l NaCl, 5 g/l yeast extract) at 30 or 37 °C with 200 rpm shaking. 30 mg/l kanamycin, 50 mg/l spectinomycin, 10 mM arabinose, or 0.5 mM isopropyl-β-d-thiogalactopyranoside (IPTG) were added when necessary [20].

**Table 1:** Bacterial strains and plasmids used in this study

Strains or plasmids	Description	Sources
Strains		
JM109	Wild-type *E. coli*	NEB
MG1655	Wild-type *E. coli* K12; F^−^ λ^−^ rph-1	CGSC 6300
TWF001	l-Threonine-producing *E. coli* strain	[21]
TSW001	TWF001△*proP*	This study
TSW002	TWF001△*proVWX*	This study
TSW003	TWF001△*proP*△*proVWX*	This study
TSW004	TWF001△*betABIT*	This study
TSW005	TWF001△*proP*△*proVWX*△*betABIT*	This study
TSW006	TWF001△*proP*△*proVWX*△*proC*	This study
TSW007	TWF001△*proP*△*proVWX*△*fadR*	This study
TSW008	TWF001△*proP*△*proVWX*△*crr*	This study
TSW009	TWF001△*proP*△*proVWX*△*ptsG*	This study
Plasmids		
pCas	*repA101*(Ts) *kan P*_*cas*_*-cas9 P*_*araB*_-*Red lacI*^q^ *P*_*trc*_ -sgRNA-*pMB1*	[27]
pTargetF	*pMB1 aadA* sgRNA	[27]
pTargetF-*proP*	*pMB1 aadA* sgRNA-*proP*	This study
pTargetF-*proVWX*	*pMB1 aadA* sgRNA-*proVWX*	This study
pTargetF-*betABIT*	*pMB1 aadA* sgRNA-*betABIT*	This study
pTargetT-*ptsG*	*pMB1 aadA* sgRNA-*ptsG*	This study
pTargetT-*crr*	*pMB1 aadA* sgRNA-*crr*	This study
pTargetT-*proC*	*pMB1 aadA* sgRNA-*proC*	This study

*E. coli* strains were inoculated to LB plates overnight at 37 °C. We used STF medium as seed medium, containing 10 g/l sucrose, 5 g/l yeast extract, 20 g/l trypton, 15 g/l (NH_4_)_2_SO_4_ and 1 g/l MgSO_4_). A small number of colonies were inoculated into 5 mL STF medium as first seed medium at 37 °C, 200 rpm for 4 h. Then inoculated the bacteria from 5 mL STF medium and added to 30 mL STF medium as second medium, keep the initial OD_600_ at 0.1, and shake again for 4 h at 37° C and 200 rpm. Finally, 5 mL of bacterial culture was added to a 500-mL fermentation flask containing 30 mL of fermentation medium (2 g/l yeast extract, 2 g/l citric acid, 25 g/l (NH_4_)_2_SO_4_, 7.46 g/l KH_2_PO_4_, 30 g/l or 40 g/l glucose, 2 g/l MgSO_4_·7 H_2_O, 5 mg/l FeSO_4_·7H_2_O, 5 mg/l MnSO_4_·4 H_2_O, and 20 g/l CaCO_3_, pH 6.8) [28]. The fermentation was performed for 36 h or 48 h at 37 ^o^C and 200 rpm, and samples were taken every 8 h.

### Construction of various plasmids

All the plasmids used in this work are listed in Table 1. All primers used in this work are listed in Table 2. The genomic DNA of *E. coli* MG1655 was used as templates for PCR amplification of the upstream and downstream fragments of the target genes. The genomic DNA and plasmids were extracted by using the TIANGEN prep kit (Tiangen, Beijing, China). The DNA polymerase 2xSuper Pfx PCR MasterMix and 2xFlashHot Start MasterMix (Dye) (CoWinbio, China) were used for PCR. Restriction endonucleases and T4 DNA ligase were purchased from Thermo Scientific (Waltham, USA) (Table 3).

**Table 2:** The primers and synthetic DNA sequences used in this study

Names	Sequence (5′–3′)
*proP*f1	TGCCGCCTTGTATGAGTG
*proP*r1	CAAATCGGCAATCTCGTGACCGAGAAACCTTGTGCC
*proP*f2	GGCACAAGGTTTCTCGGTCACGAGATTGCCGATTTG
*proP*r2	CCCATTGCCATTCACAGC
*proP*-sgRAN-F	GCATTACTCCGAAGACCACGGTTTTAGAGCTAGAAATAGC
sgRNA-R	AGTATTATACCTAGGACTGAGCTAG
Y-pTargetF-F	CGTATTACCGCCTTTGAGTGAG
Y-pTargetF-R	GCTTATGGAGCTGCACATGAACT
*proVWX*f1	AGCAGTCCACGGTTACTACAT
*proVWX*r1	GCCCTCTTTGTAGCGACTTTCCGGCCAATTCCATACC
*proVWX*f2	GTATGGAATTGGCCGGAAAGTCGCTACAAAGAGGGC
*proVWX*r2	TCAAACATATCACCGAGGG
*proVWX*-sgRAN-F	ACGACTGCACCGACTGACGGGTTTTAGAGCTAGAAATAGC
*betABIT*f1	ATTGAACATTCGCAACAGC
*betABIT*r1	CGGACATCTCGTCGTAACCCTACAGCAAAGAGCAGGTGAT
*betABIT*f2	ATCACCTGCTCTTTGCTGTAGGGTTACGACGAGATGTCCG
*betABIT*r2	TGGCGTTGTCTGGTTTATTT
*betABIT*-sgRAN-F	ATCTACCCACCGATTAACCGGTTTTAGAGCTAGAAATAGC
*proC*f1	CCTTTGTTGGGTAATCCTCT
*proC*r1	CCATACACTTCGTCATCGCTGTCTTTATTCAGGCTGGAGG
*proC*f*2*	CCTCCAGCCTGAATAAAGACAGCGATGACGAAGTGTATGG
*proC*r2	CGGTAGCGATGTGATTGG
*proC*-sgRAN-F	GATATGGTCTGCTCACCGGGGTTTTAGAGCTAGAAATAGCTAGC
RT-16sRNA-F	TCGGGAACCGTGAGACAGG
RT-16sRNA-R	CCGCTGGCAACAAAGGATAAG
RT-*glk*-F	CTTGCTCTGTGTGATATTGC
RT-*glk*-R	GTCATCGCCACCCAGTCAC
RT-*pgi*-F	GTTCTGGGACTGGGTTGGCG
RT-*pgi*-R	GCGTGGTGGAGAAATGCTTG
RT-*zwf*-R	TAAACGTCTGCCGACCAAAT
RT-*zwf*-R	AGATGCGTCTGATTAAAGGT
RT-*ptsG*-F	TTTTGTTGTAATGCGGTGGTT
RT-*ptsG*-R	GCAGGGAAAGTGTGTTGTCG
RT-*crr*-F	AACCCTGAAGAACTGATTGGC
RT-*crr*-R	GCACCGCAACTTCACTCTTC
RT-*galP*-F	CACAACAAGTCCAGCA TTCC
RT-*galP*-R	AGCCGCACCTTTACCATC
RT-*mglB*-F	TGAAGAAA TCGCCTCTAAAC
RT-*mglB*-R	GCAAAGAAGCCAAGA TACTC
RT-*ndh*-F	TGGGACGCAAGAAAAAAGC
RT-*ndh*-R	CACGATCAATATCAATGACGG
RT-*betA*-F	GCGTCACTACCTCCAAACCC
RT-*betA*-R	TCCATCGGACCAAAACCTTC
RT-*betB*-F	ACTCGCAAAACTGGAAACCC
RT-*betB*-R	GCAATCTGGATCGGGTAGTTC
RT-*betT*-F	CACCTGCTCTTTGCTGTAGTCC
RT-*betT*-R	CAGTTGGGTCATCTGGATTTGT

**Table 3:** Comparison of fermentation parameters of different strains in flask fermentation

Strains	Glucose (g/l)	Titer (g/l)	Productivity (g/l/h)	Yield on glucose (g/g)
TWF001	30	12.3 ± 0.1	0.342 ± 0.003	0.410 ± 0.003
	40	16.5 ± 1.0	0.458 ± 0.028	0.412 ± 0.025
TSW001	30	17.6 ± 0.7	0.489 ± 0.019	0.587 ± 0.023
	40	21.0 ± 0.5	0.583 ± 0.014	0.525 ± 0.013
TSW002	30	15.7 ± 0.3	0.436 ± 0.008	0.523 ± 0.010
	40	21.1 ± 1.0	0.586 ± 0.028	0.528 ± 0.025
TSW003	30	19.0 ± 0.5	0.528 ± 0.014	0.633 ± 0.017
	40	24.1 ± 0.9	0.669 ± 0.025	0.603 ± 0.023
TSW004	30	13.5 ± 0.2	0.375 ± 0.006	0.450 ± 0.007
	40	15.6 ± 0.1	0.433 ± 0.003	0.390 ± 0.003
TSW005	30	14.9 ± 2.0	0.414 ± 0.056	0.497 ± 0.067
	40	13.3 ± 2.0	0.369 ± 0.056	0.333 ± 0.050
TSW006	30	17.2 ± 0.3	0.573 ± 0.008	0.573 ± 0.010
	40	17.7 ± 0.1	0.492 ± 0.003	0.443 ± 0.003
TSW007	30	18.0 ± 0.3	0.500 ± 0.008	0.600 ± 0.01
	40	18.1 ± 0.5	0.503 ± 0.014	0.453 ± 0.013
TSW008	30	19.7 ± 0.4	0.547 ± 0.011	0.657 ± 0.013
	40	25.0 ± 1.3	0.694 ± 0.036	0.625 ± 0.033
TSW009	30	19.6 ± 0.5	0.544 ± 0.014	0.653 ± 0.017
	40	26.0 ± 2.2	0.542 ± 0.046	0.650 ± 0.055

The plasmid pTargetF-*proP* was constructed from pTargetF by inverse PCR, using the primers *proP*-sgRNA-F and sgRNA-R, and self-ligation. Its sequence was confirmed by sequencing, using primers Y-pTargetF-F and Y-pTargetF-R. Similarly, pTargetF-*proVWX* was constructed using the primer *proVWX*-sgRNA-F instead of *proP*-sgRNA-F, pTargetF-*betABIT* was constructed using the primers *betABIT*-sgRNA-F, and pTargetF-*proC* was constructed using the primers *proC*-sgRNA-F.

### Construction of *E. coli* mutant strains TSW001, TSW002, TSW003 and TSW004

The gene deletion in the chromosome of *E. coli* strains was undertaken according to the published method [27].

**Fig. 1:**
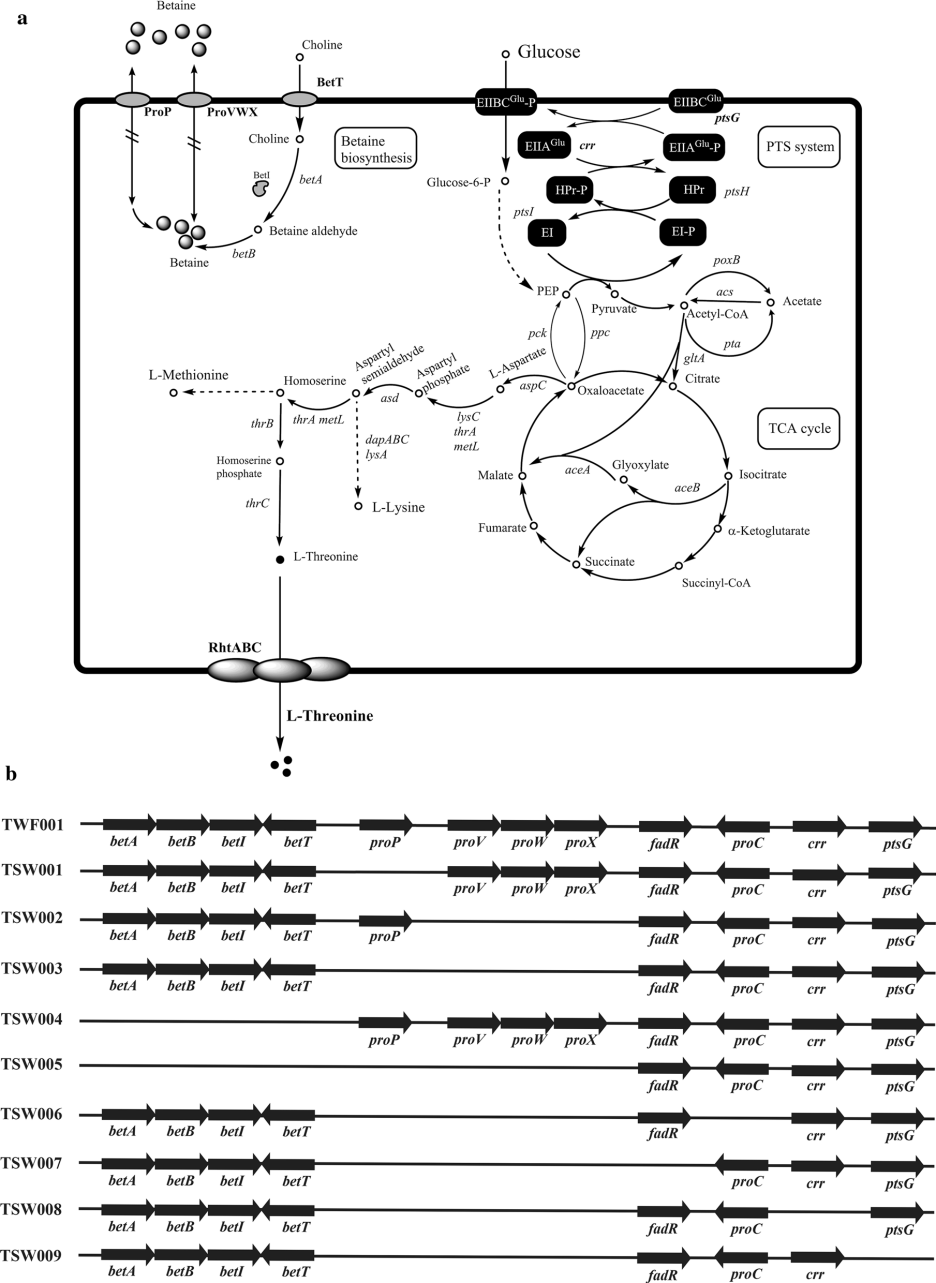
**a** The pathways and respective enzymes or genes to show the metabolic engineering strategy applied to create recombinant *E. coli* strains in this study. Osmosensing transporters ProP and ProVWX are responsible for the transport of betaine and proline. BetA and BetB are responsible for the synthesis of betaine, and BetT is driven by proton-motive-force to transport the betaine precursor choline into the cell. BetI regulates the expression of bet operon. EIIA^Glc^ encoded by *crr* and EIIBC^Glc^ encoded by *ptsG* are key enzymes in the phosphotransferase system. **b** The comparison of mutations on the chromosome of different *E. coli* strains used in this study

TSW001 was constructed from TWF001 by deleting *proP* (Fig. 1). The pCas was transformed into TWF001, resulting TWF001/pCas. Arabinose was used to activate the expression of Red enzymes for recombination in TWF001/pCas [27]. Primers *proP*f1 and *proP*r1 were used to amplify the upstream fragment of *proP*, while primers *proP*f2 and *proP*r2 were used to amplify the downstream fragment of *proP*. These fragments were overlapped by using primer *proP*f1 and *proP*r2. 500 ng of the overlapped fragment and 100 ng of pTargetF-*proP* were mixed with 100 µL TWF001/pCas competent cells, and electroporated. Cells were recovered for 1 h at 30 °C, spread onto LB agar plate containg spectinomycin and kanamycin, and then incubated at 30 °C for 24~36 h. The correct transformants were confirmed by colony PCR using primer *proP*f1 and *proP*r2. In the end, pTargetF-*proP* was cured by adding IPTG, and then pCas was cured by growing at 42 °C for 24 h.

Similarly, TSW002 was constructed from TWF001 by deleting *proVWX* (Fig. 1). Primers *proVWX*f1 and *proVWX*r1 were used to amplify the upstream fragment, while primers *proVWX*f2 and *proVWX*r2 were used to amplify the downstream fragment. These fragments were overlapped and the correct transformants were confirmed by by using primer *proVWX*f1 and *proVWX*r2.

TSW003 was constructed from TSW001 by deleting *proVWX* (Fig. 1). Primers *proVWX*f1 and *proVWX*r1 were used to amplify the upstream fragment, while primers *proVWX*f2 and *proVWX*r2 were used to amplify the downstream fragment. These fragments were overlapped and the correct transformants were confirmed by using primer *proVWX*f1 and *proVWX*r2.

TSW004 was constructed from TWF001 by deleting *betABIT* (Fig. 1). Primers *betABIT*f1 and *betABIT*r1 were used to amplify the upstream fragment of *betABIT*, while primers *betABIT*f2 and *betABIT*r2 were used to amplify the downstream fragment. These fragments were overlapped and the correct transformants were confirmed by using primer *betABIT*f1 and *betABIT*r2.

### Construction of ***E. coli*** mutant strains TSW005, TSW006, TSW007, TSW008 and TSW009

TSW005 was constructed from TSW003 by deleting *betABIT* (Fig. 1). Primers *betABIT*f1 and *betABIT*r1 were used to amplify the upstream fragment of *betABIT*, while primers *betABIT*f2 and *betABIT*r2 were used to amplify the downstream fragment. These fragments were were overlapped and the correct transformants were confirmed by by using primer *betABIT*f1 and *betABIT*r2. TSW006 was constructed from TSW003 by deleting *proC* (Fig. 1). The pCas was transformed into TSW003. Primers *proC*f1 and *proC*r1 were used to amplify the upstream fragment of *proC*, while primers *proC*f2 and *proC*r2 were used to amplify the downstream fragment of *proC*. These fragments were overlapped and the correct transformants were confirmed by using primer *proC*f1 and *proC*r2.

TSW007 was constructed from TSW003 by deleting *fadR* (Fig. 1). The pCas was transformed into TSW003. The activated TSW003/pCas made competent cells. Primers *fadR*f1 and *fadR*r1 were used to amplify the upstream fragment of *fadR*, while primers *fadR*f2 and *fadR*r2 were used to amplify the downstream fragment of *fadR*. These fragments were overlapped and the correct transformants were confirmed by using primer *fadR*f1 and *fadR*r2.

TSW008 was constructed from TSW003 by deleting *crr* (Fig. 1). The activated TSW003/pCas made competent cells. Primers *crr*f1 and *crr*r1 were used to amplify the upstream fragment of *crr*, while primers *crr*f2 and *crr*r2 were used to amplify the downstream fragment of *crr*. These fragments were overlapped and the correct transformants were confirmed by using primer *crr*f1 and *crr*r2.

TSW009 was constructed from TSW003 by deleting *ptsG* (Fig. 1). Primers *ptsG*f1 and *ptsG*r1 were used to amplify the upstream fragment of *ptsG*, while primers *ptsG*f2 and *ptsG*r2 were used to amplify the downstream fragment of *ptsG*. These fragments were overlapped and the correct transformants were confirmed by using primer *ptsG*f1 and *ptsG*r2.

### Analysis of cell growth, glucose and l-threonine concentration

Biomass was characterized by the OD_600_ value measured by the UV-1800 spectrophotometer (Shimadzu, Japan). To check the levels of l-threonine and glucose, 800 µL culture was taken and centrifugated 20 min at 13,800 g to obtain the supernatant. 10 µL supernatant was diluted 100 times to determine the residual glucose in the medium, using the SBA-40E biosensor (Shandong, China). 50 µL of the supernatant was diluted with trichloroacetic acid (for protein precipitation) to 1 mL to determine the l-threonine content in the medium, using the 1260 series High Performance Liquid Chromatography (HPLC) system (Agilent Technology, USA). Thermo ODS-2HYPERSIL C18 column (250 mm × 4.0 mm, USA) was used to separate and quantify l-threonine, using the orthophthalaldehyde precolumn derivatization method [20]. The mobile phases used are solvent mixtures A and B. Solvent A contains 3.01 g sodium acetate, 200 µL triethylamine and 5 mL tetrahydrofuran per liter, and solvent B were prepared by mixing 3.01 g sodium acetate, 400 mL acetonitrile and 400 mL methanol in 200 mL deionized water. The two solvents were adjusted to a flow rate of 0.8 mL/min from 8% of solvent B to 100% in 18 min. 20 µL sample was derivatized, injected and monitored at 338 nm.

### Quantification of mRNA by real‐time PCR

The transcriptional levels of *betA*, *betB*, *betT*, *pgi*, *galP*, *mglB*, *glk*, *zwf*, *pstG*, *crr* and *ndh* in TWF001 and TSW003 was quantified using the real-time PCR (RT-PCR). Total RNA was extracted from TSW003 and TWF001 grown at late logarithmic. Total RNA was extracted using the Simply P Total RNA Extraction Kit (Biofluorescence, Beijing, China; BSC52S1). The mass of total RNA was quantified by spectrophotometry (nanodrops, Thermal Sciences, Shanghai, China) and electrophoresis respectively. Genomic DNA elimination and cDNA synthesis were performed using the HiScript first-strand cDNA synthesis kit for quantitative polymerase chain reaction (Vazyme, Nanjing, China; R312-01). ChamQ Universal SYBR qPCR master mix (Vazyme, Nanjing, China; Q711-02) and the ABI First Step Quantitative Polymerase Chain Reaction System (Applied Biosystems, San Mateo, CA) were used to perform quantitative polymerase chain reactions under the following reaction conditions: 95 °C for 3 min, followed by 40 cycles of 95 °C for 10 s, 56 °C for 30 s, and 72 °C for 30 s. The primers used in the qPCR reaction are listed in Table 2. As an internal standard control, the relative abundance of 16S rRNA was used to standardize the results. All assays were performed in triplicate.

## Results

### Addition of betaine slightly improved l-threonine production in ***E. coli*** TWF001

**Fig. 2:**
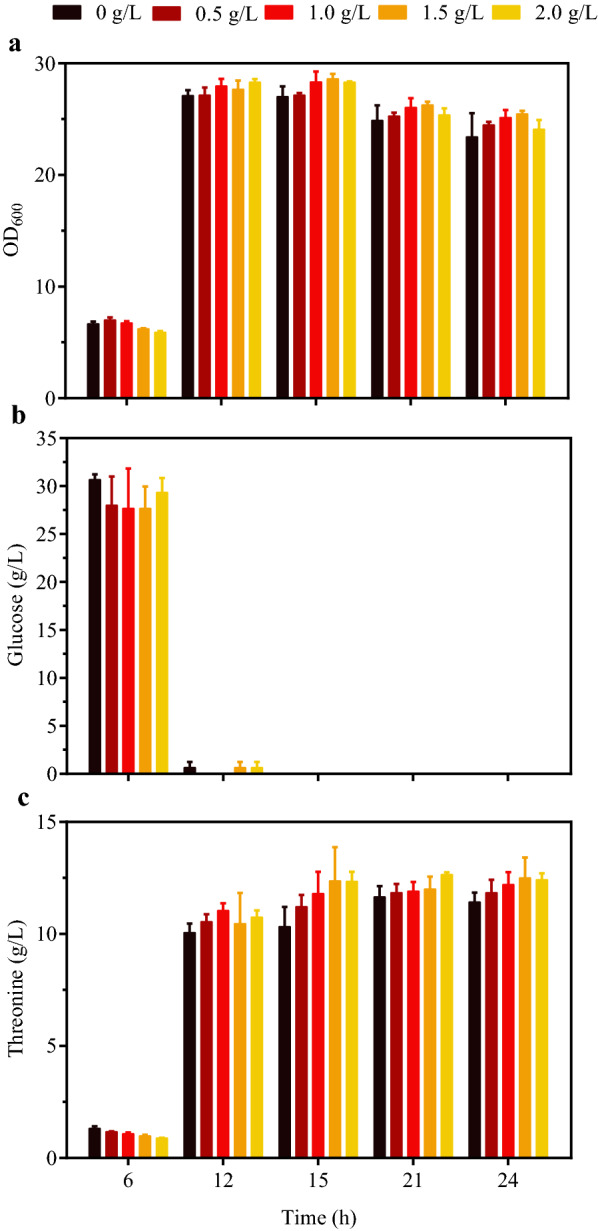
Fermentation of *E. coli* TWF001 with the addition of betaine. **a** Cell growth; **b** Glucose consumption; **c** l-threonine production. The error bars indicate the standard deviations from three independent experiments

l-Threonine producing *E. coli* strain TWF001 can produce 11.2 g/l l-threonine from 30 g/l glucose after 24 h flask-fermentation [21] (Fig. 2). To investigate the influence of betaine on l-threonine production of TWF001, 0.5, 1.0, 1.5 and 2.0 g/l betaine were added during fermentation [4], the cell growth, glucose consumption, and l-threonine production were determined and compared (Fig. 2). Without the addition of betaine, TWF001 produced 11.5 g/l l-threonine from 30 g/l glucose after 24 h. The addition of betaine only slightly increased l-threonine production in TWF001. The highest l-threonine production (12.5 g/l) was obtained with the addition of 1.5 g/l betaine. In fact,12.3 g/l l-threonine has been produced after 15 h in TWF001 with the addition of 1.5 g/l betaine (Fig. 2c). This is understandable because after 15 h the glucose has been completely consumed (Fig. 2b) and the cells stopped to grow (Fig. 2a). The results suggest that the addition of betaine can influence the production of l-threonine in TWF001 possibly due to the change of osmotic pressure.

### Deletion of ***proP*** and ***proVWX*** significantly improved l-threonine production in *E. coli* TWF001

**Fig. 3:**
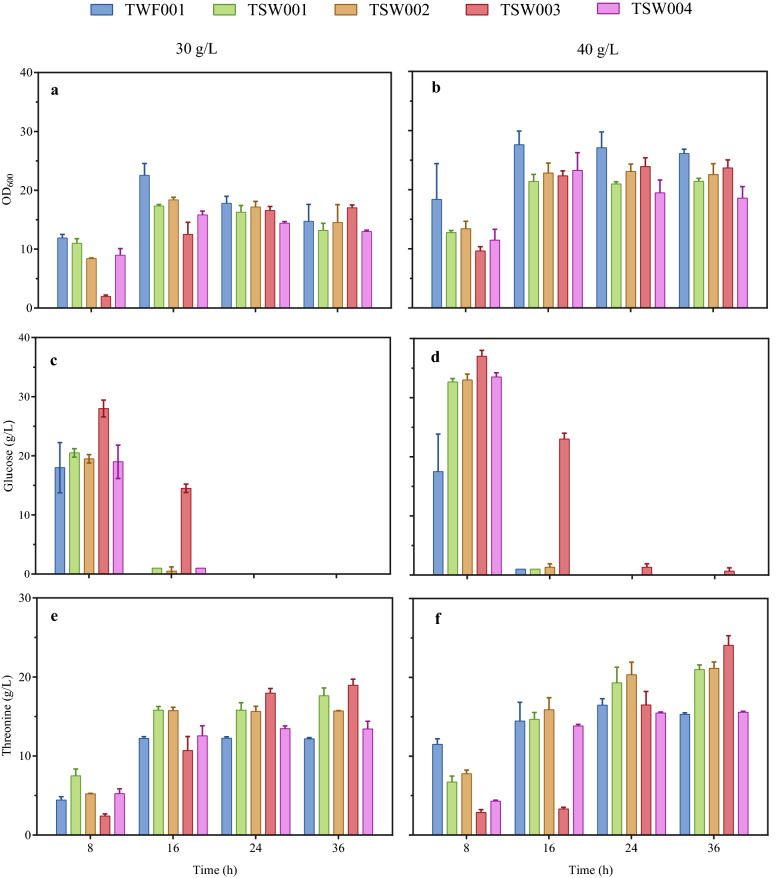
Fermentation of *E. coli* TWF001, TSW001, TSW002, TSW003, and TSW004 with 30 g/l or 40 g/l initial glucose. **a** Cell growth, 30 g/l glucose; **b** Cell growth, 40 g/l glucose; **c** Glucose consumption, 30 g/l glucose; **d** Glucose consumption, 40 g/l glucose; **e** l-threonine production, 30 g/l glucose; **f** l-threonine production, 40 g/l glucose. The error bars indicate the standard deviations from three independent experiments

To further investigate the influence of osmotic pressure on l-threonine production in TWF001, several genes (*proP*, *proVWX* and *betABIT*) related to osmoregulation were deleted, resulting the mutant strains TSW001, TSW002, TSW003 and TSW004 (Fig. 1). Compared with the control TWF001, the mutant strains TSW001, TSW002 and TSW004 always grew slower, consumed glucose slower, but produced more l-threonine during 36 h fermentation with either 30 or 40 g/l initial glucose (Fig. 3). The highest l-threonine production (21.1 g/l) was obtained in TSW002 after 36 h fermentation with 40 g/l initial glucose (Fig. 3f). The patterns of cell growth, glucose consumption and l-threonine production in TSW003 in which *proP* and *proVWX* were deleted were quite different from others. Compared with the control TWF001, TSW003 grew significantly slower with 30 g/l initial glucose (Fig. 3a), consumed glucose significantly slower (Fig. 3c), and produced less l-threonine at the earlier stage of fermentation, however, at the later stage TSW003 gradually grew better and produced more l-threonine (Fig. 3). After 36 h fermentation, TSW003 produced 18.9 g/l l-threonine under 30 g/l initial glucose (Fig. 3e) with a glucose conversion rate of 0.63 g/g, and produced 23.5 g/l l-threonine under 40 g/l initial glucose (Fig. 3f) with a glucose conversion rate of 0.59 g/g.

**Fig. 4:**
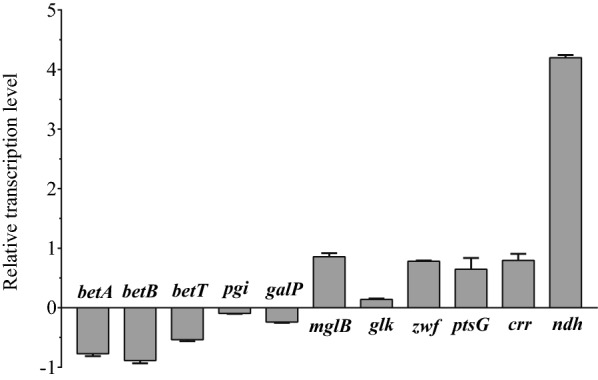
Relative transcription levels of *betA*, *betB*, *betT*, *pgi*, *galP*, *mglB*, *glk*, *zwf*, *ptsG*, *crr* and *ndh* in TSW003, using TWF001 as a control. The error bars indicate the standard deviations from three independent experiments

To understand the higher l-threonine production in TSW003, transcriptional levels of some key genes related to betaine synthesis (*betA*, *betB*, and *betT*) and glycolysis (*pgi*, *galP*, *mglB*, *glk*, *zwf*, *pstG*, *crr* and *ndh*) were determined by qPCR (Fig. 4). Compared with the control TWF001, *betA*, *betB* and *betT* in TSW003 were down-regulated, suggesting that the absence of betaine transporters ProP and ProVWX somehow down-regulates the intracellular betaine biosynthesis. Glycolysis and glucose absorption are important metabolic pathways, therefore, the transcriptional levels of *pgi*, *galP*, *mglB*, *glk*, *zwf*, *pstG*, *crr* and *ndh* were determined. Compared with the control TWF001, the galactose transporter (*galP*) was slightly down-regulated, while the galactose and glucose transporter (*mglB*), the key genes *ptsG* and *crr* in the PTS system were up-regulated. As to the key genes in the glycolysis pathway, there was almost no change for glucose-6 phosphate isomerase (*pgi*) and glucokinase (*glk*), but glucose-6-phosphate dehydrogenase (*zwf*) was up-regulated. The results suggest that when *proP* and *proVWX* are knocked out in *E. coli*, the intracellular osmotic pressure was disturbed, the cells might increase the uptake and utilization of carbon sources to maintain normal physiological activities. In addition, NADH dehydrogenase (*ndh*) was significantly up-regulated in TSW003, suggesting that enough energy might play an important role on higher l-threonine production in TSW003.

### Metabolic engineering of TSW003 to further increase l-threonine production

**Fig. 5:**
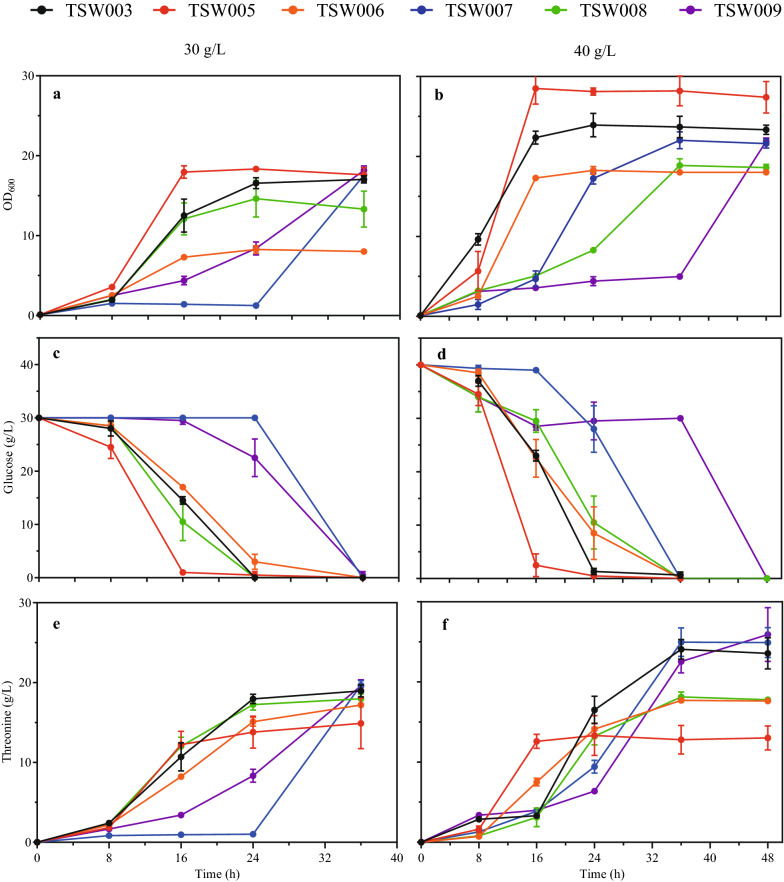
Fermentation of TSW003, TSW005, TSW006, TSW007, TSW008, and TSW009 with 30 g/l or 40 g/l initial glucose. **a** Cell growth, 30 g/l glucose; **b** Cell growth, 40 g/l glucose; **c** Glucose consumption, 30 g/l glucose; **d** Glucose consumption, 40 g/l glucose; **e** l-threonine production, 30 g/l glucose; **f** l-threonine production, 40 g/l glucose. The error bars indicate the standard deviations from three independent experiments

TSW005 was constructed from TSW003 by deleting the gene cluster *betABIT*, TSW006 was constructed from TSW003 by deleting *proC*, and TSW007 was constructed from TSW003 by deleting *fadR* (Fig. 1). Compared to TSW003, TSW005 grew better (Fig. 5a) but produced less l-threonine under 30 g/l initial glucose (Fig. 5e), TSW006 and TSW007 grew worse (Fig. 5a) and produced less l-threonine (Fig. 5e). TSW008 was constructed from TSW003 by deleting the gene *crr* encoding enzyme IIA (EIIA^Glc^), and TSW009 was constructed from TSW003 by deleting the gene *ptsG* encoding enzyme EIIBC^Glc^. Under the initial glucose of 30 g/l, TSW008 and TSW009 hardly consumed glucose in the first 24 h (Fig. 5c), started to grow after 24 h (Fig. 5a), and the final l-threonine production reached 19.6 g/l after 36 h fermentation (Fig. 5e).

When the initial glucose increased from 30 to 40 g/l, similar patterns of growth, glucose consumption and l-threonine production in TSW005, TSW006, TSW007, TSW008 and TSW009 were observed (Fig. 5). The highest l-threonine production was obtained in TSW008 after 36 h fermentation (25.0 g/l) with a productivity of 0.69 g/l/h or in TSW009 after 48 h fermentation (25.9 g/l) with a productivity of 0.54 g/l/h. The results indicate that deletion of *crr* or *ptsG* can enhance l-threonine production by saving phosphoenolpyruvate but cause severe growth retardation due to the damage on glucose uptake system.

## Discussion

Betaine has always been an important additive in microbial fermentation engineering. The current research on the fermentation process mainly focuses on betaine addition. After 24 h fermentation supplemented with betaine l-threonine titer increased 13.3% in *E. coli* THRD [3]. As for the mechanism of adding betaine to improve the fermentation properties of strains, betaine increases the transcription level of the *zwf* encoding glucose-6-phosphate dehydrogenase [3]. Addition of betaine can protect the protein activity in the cell when the environmental osmotic pressure rises [29, 30] and maintains the ion balance inside and outside the cell [31]. Compatible solutes are thought to relieve osmotic stress by maintaining preferential exclusion of the proteins that would otherwise be affected by ions such as potassium [18]. Betaine can increase the survival rate of microbial cells during dehydration and rehydration during the fermentation process, protect the activity of various enzymes in the cell, and sometimes act as a methyl groups donor, the unique bio-compatibility and physiological activity of betaine will make it have a broad application prospect in the field of biotechnology.

In this study, the l-threonine-producing strain TSW008 and TSW009 improved the synthesis pathway of *E. coli* l-threonine by changing the intracellular osmotic pressure and accumulating the osmotic protectant betaine, as well as regulating sugar intake. We knocked out genes related to betaine synthesis and transport in *E. coli* to obtain a highly l-threonine-producing modified strain TSW003. The increase in the transcription level of the glucose uptake system and the enhancement of NADH synthesis were used to explain the mechanism of increased l-threonine production after knocking out *proP* and *proVWX.* Futher deletion of *betABIT* and *proC* on TSW003 indicated that in the transport of betaine and proline mediated by the *proP* and *proVWX* systems, after losing the intracellular synthesis of betaine, the cells are unable to synthesis betaine completely, and the cells may not use the characteristics of betaine to increase biomass and production. Because proline is also involved in intracellular protein and other metabolism, unlike betaine, which has a single function, it may be the reason for the growth rate and the production of l-threonine did not decrease significantly like TSW005. The results proved the unique effect of betaine on cell growth and l-threonine production during fermentation. And compared to the previous work which knockout of *crr* and *ptsG* alone [26], we knocked out the above two genes on TSW003, which did not get a greatly increase the yield and caused serious growth slowdown. This may be related to the dependence of cells on the PTS system after knocking out *proP* and *proVWX*, which demonstrated in the reverse transcription PCR experiment.

As an important carbon source material for metabolism, glucose has a wide range of applications and can provide the energy needed for the growth and reproduction of bacteria. Glucose is transported from the external environment to the cytoplasm through cell membrane, and then metabolism occurs in the cytoplasm. The concentration of glucose in the external environment is not always balanced with that in the cytoplasm. In order to adapt to the environment, bacteria have evolved to form different mechanisms to transport substances into or out of the cell. Glucose concentration is closely related to changes in environmental osmotic pressure during fermentation. High concentration of glucose usually retards cell growth. Osmotic pressure-deficient strains promote the utilization of glucose, and some genes encoding membrane proteins are sensitive to glucose metabolism [32]. When the osmotic pressure changed and can no longer maintain the stability of intracellular osmotic pressure through self-regulation, *E. coli* might increase the carbon sources utilization to rebalance its osmotic pressure.

In addition to changes in glucose utilization, the biosynthesis of some amino acids in bacteria is also affected by their own or external osmotic pressure. Under osmotic pressure, *E. coli* can regulate the amino acid metabolic network [33]. As the last gene in methionine synthesis, *metE* works with *metH* to convert l-cysteine into methionine. A bacterial salt sensor reproduces the phenotype in GroE-deficient *E. coli*. After GroE was deleted, the *metE* promoter responds to salt concentration [34]. It has been found that acetic acid-mediated growth inhibition of *E. coli* is the result of the accumulation of homocysteine, which is a substrate of cobalamin-independent methionine synthase (MetE) which catalyzes the last step of methionine biosynthesis. The MetE enzyme was randomly mutated to increase the resistance of *E. coli* to acetic acid [35]. The leucine, isoleucine and valine transproter-1 (LIV-1) system acts as a branched-chain amino acid ABC transporter, responsible for the transport of leucine, isoleucine and valine, and the low-affinity transport of l-threonine [36], The LIV-1 system is sensitive to osmotic shocks. When cells are subjected to an osmotic shock, the activity of the LIV-1 system decreases, while the LIV-II system remains unaffected in *Pseudomonas aeruginosa* and *E. coli* [37–39]. Therefore, intracellular amino acid metabolism may change when cells encounter high external osmotic pressure or genes responsible for balancing osmotic pressure are damaged. This phenomenon may be applied to produce a variety of amino acids in fermentation engineering. Intracellular sensors in response to changes in osmotic pressure can be used to control metabolic changes to obtain target products or reduce by-products. These biosensors could be found from the osmotic related genes such as osmoprotectants transporters or osmotic pressure-responsive channel protein on the outer membrane.

In *E. coli*, phospholipid membranes are highly permeable to water but not to polar solutes. Thus, abrupt changes in external solute concentration cause water to rapidly leave or enter cells. Well defined osmoregulatory systems respond by mediating solute synthesis or the uptake of exogenous osmolytes to restore cellular hydration. Osmosensing transporters like ProP and ProVWX activate to mediate osmolyte uptake as other transporters inactivate at high osmotic pressure [40]. It showed that *E. coli* protein ProP is both an osmosensor and an osmoprotectant transporter [41]. Therefore, ProP also plays an important role in the structure of membrane wall in *E. coli*. The concentration of *E. coli* cell bipolar permeability transporter ProP increases with the increase of cardiolipin content and depends on the structure of the cytoplasmic carboxyl terminal domain of the transporter [42]. Increasing the expression of phosphatidylethanolamine synthase was found to significantly increase both the tolerance and production of octanoic acid, a representative membrane-damaging solvent. Tolerance of other industrially-relevant inhibitors, such as furfural, acetate, toluene, ethanol and low pH was also increased [43]. The increase in l-threonine production may be related to changes in membrane phospholipid composition, changes in the outer membrane of cells caused by osmotic pressure may lead to an increase or decrease in cell tolerance during fermentation, which belongs to the category of membrane engineering.

This study demonstrated that l-threonine production in *E. coli* could be efficiently increased by regulating the intracellular osmotic pressure. Further work is required to fully elucidate the relationships among amino acid fermentation, membrane wall structure and osmoregulatory transport of betaine in *E. coli*.
